# Destabilization of the TAR hairpin leads to extension of the polyA hairpin and inhibition of HIV-1 polyadenylation

**DOI:** 10.1186/1742-4690-6-13

**Published:** 2009-02-11

**Authors:** Martine M Vrolijk, Alex Harwig, Ben Berkhout, Atze T Das

**Affiliations:** 1 Laboratory of Experimental Virology, Department of Medical Microbiology, Center for Infection and Immunity Amsterdam (CINIMA), Academic Medical Center, University of Amsterdam, Meibergdreef 15, 1105 AZ Amsterdam, The Netherlands

## Abstract

**Background:**

Two hairpin structures that are present at both the 5' and 3' end of the HIV-1 RNA genome have important functions in the viral life cycle. The TAR hairpin binds the viral Tat protein and is essential for Tat-mediated activation of transcription. The adjacent polyA hairpin encompasses the polyadenylation signal AAUAAA and is important for the regulation of polyadenylation. Specifically, this RNA structure represses polyadenylation at the 5' side, and enhancer elements on the 3' side overcome this suppression. We recently described that the replication of an HIV-1 variant that does not need TAR for transcription was severely impaired by destabilization of the TAR hairpin, even though a complete TAR deletion was acceptable.

**Results:**

In this study, we show that the TAR-destabilizing mutations result in reduced 3' polyadenylation of the viral transcripts due to an extension of the adjacent polyA hairpin. Thus, although the TAR hairpin is not directly involved in polyadenylation, mutations in TAR can affect this process.

**Conclusion:**

The stability of the HIV-1 TAR hairpin structure is important for the proper folding of the viral RNA transcripts. This study illustrates how mutations that are designed to study the function of a specific RNA structure can change the structural presentation of other RNA domains and thus affect viral replication in an indirect way.

## Background

**Figure 1:**
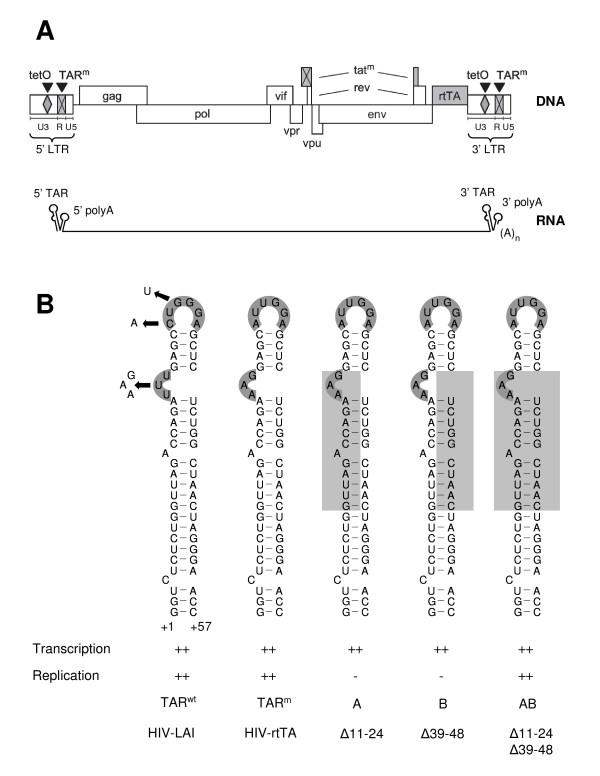
**The HIV-rtTA genome and mutations in the TAR hairpin**. (A) The HIV-rtTA proviral DNA genome and the viral RNA transcript are shown. In this virus the Tat-TAR axis of transcription regulation was inactivated by mutation of both Tat and TAR (tat^m ^and TAR^m^; crossed boxes) and functionally replaced by the doxycycline(dox)-inducible Tet-ON gene regulation system [32,33]. The tetO elements were introduced in the U3 promoter region and the Nef gene was replaced by the rtTA gene. The R region that is present at both the 5' and 3' end of the viral transcript folds the TAR and polyA hairpin elements. The latter structure is truncated upon polyadenylation at the 3' R. (B) The wild-type TAR hairpin (TAR^wt^) and the TAR^m ^version with bulge and loop mutations as present in the HIV-rtTA virus are shown. The TAR^m ^sequence is partially deleted in the mutants A, B and AB. The deleted nucleotides are indicated by a grey box. The transcription and replication properties of these mutant viruses are indicated as previously presented [34,35].

All retroviral RNA genomes contain a repeat (R) region at the extreme 5' and 3' end. This sequence repeat allows the first strand transfer step of the reverse transcription process, which results in the formation of long terminal repeat (LTR) regions in the proviral DNA. The 97-nt R region in HIV-1 RNA can fold two stem-loop structures, the TAR and polyA hairpins (Fig. 1A). Both motifs have important functions in the biosynthesis of viral transcripts. The TAR hairpin contains a highly conserved 3-nucleotide pyrimidine bulge that binds the viral Tat transactivator protein [1] and an apical 6-nucleotide loop that binds the cyclin T1 subunit of the cellular transcriptional elongation factor (pTEFb) in a Tat-dependent manner [2-4]. The TAR bound CDK9 kinase component of pTEFb phosphorylates the C-terminal domain of RNA polymerase II, which enhances the processivity of the elongating polymerase [5,6]. Furthermore, it was demonstrated that pTEFb directs the recruitment of TATA-box-binding protein (TBP) to the LTR promoter to stimulate the assembly of new transcription complexes [7,8]. In addition to its role in transcription, the TAR hairpin has been suggested to be important for dimerization of the viral RNA genome [9], packaging of the viral RNA into virions [10-14], the strand transfer step of reverse transcription [15], and as a possible HIV-1 derived miRNA with a role in latency [16,17].

The polyA hairpin encompasses the AAUAAA polyadenylation signal that is recognized by the cleavage polyadenylation specificity factor (CPSF), resulting in polyadenylation of the viral transcripts. Whereas TAR should exert its function in the 5' LTR promoter context, the polyadenylation signal should be recognized exclusively in the 3' LTR context. Previous studies indicated that usage of the 3' polyadenylation site is promoted by an upstream sequence element (USE) in the U3 region that is uniquely present at the 3' end of viral transcripts [18-22]. This element enhances binding of CPSF to the AAUAAA motif [23]. The 5' polyadenylation site may also be inactive because it is positioned close to the transcription initiation site, such that polyadenylation factors have not yet gained access to the nascent transcript through the RNA polymerase II complex [24-26]. Moreover, binding of U1 snRNP to the major splice donor site that is uniquely present downstream of 5' R represses polyadenylation at the 5' polyadenylation signal [27,28]. We previously demonstrated that the polyA hairpin masks the AAUAAA signal from recognition by CPSF and that the stability of the polyA hairpin is delicately balanced to allow 5' repression and 3' activation of polyadenylation [29-31].

We recently used the designed HIV-rtTA variant that does not need TAR for activation of transcription (Fig. 1A)[32,33] to study additional functions of TAR in viral replication by deleting parts of this motif [34]. We observed that virus mutants with a deletion on either the left or right side of the TAR stem (mutants A and B in Fig. 1B, respectively) are replication deficient, whereas the double mutant with a truncated TAR stem (AB) and variants with a complete TAR deletion replicate efficiently. This latter result indicates that TAR has no essential function in the viral life cycle other than to accommodate Tat-mediated activation of transcription. To understand why the single-side deletions abolished replication, we previously analyzed the effect of these mutations on the HIV-1 RNA structure [35]. These assays with *in vitro *produced transcripts revealed that the 5' TAR-destabilizing mutations affect the proposed riboswitch of the leader RNA, the so called LDI-BMH equilibrium [36-38]. Whereas the wild-type transcript adopts predominantly the LDI conformation, the A and B mutants demonstrate a shift toward the alternative BMH conformation. As a result, the DIS hairpin that mediates RNA dimerization is more exposed, which affects dimerization [35] and packaging of the transcripts into virion particles (unpublished results). We now demonstrate an effect of 3' TAR destabilization on 3' polyadenylation of the viral transcripts *in vivo*. We propose that unpaired TAR nucleotides extend the polyA hairpin, thus restricting the availability of the AAUAAA signal for CPSF binding and polyadenylation.

## Results

### HIV-rtTA expression is reduced by destabilization but not by truncation of the TAR hairpin

**Figure 2:**
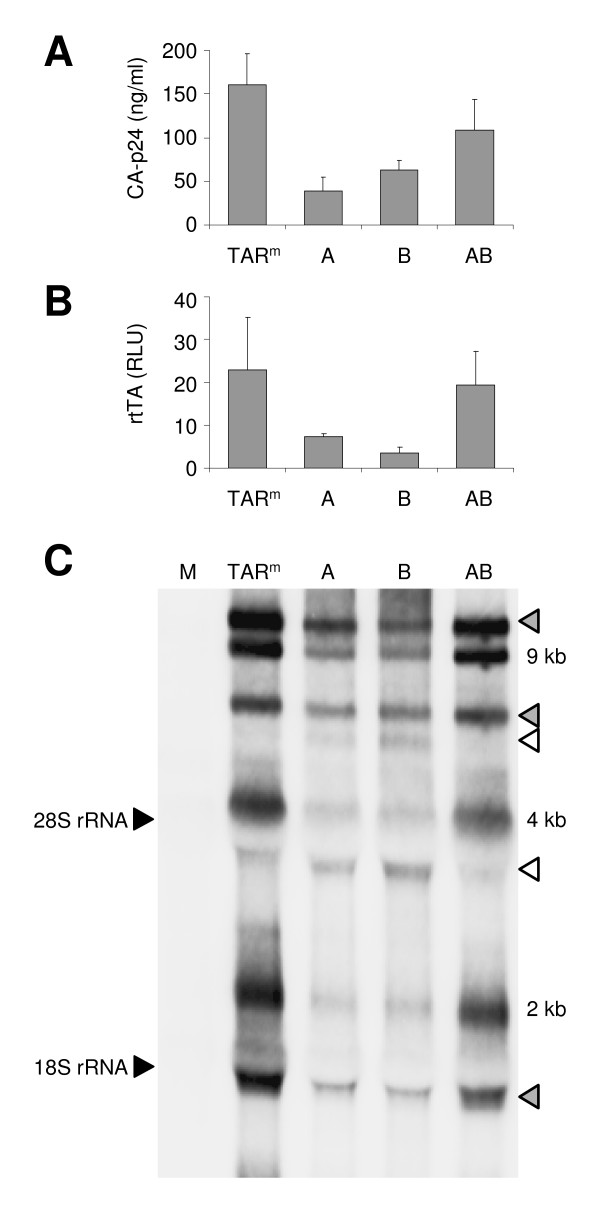
**Destabilization of the TAR hairpin affects viral gene expression**. (A) C33A cells were transfected with the original (TAR^m^) and TAR-deleted HIV-rtTA variants (mutants A, B and AB) and cultured with dox. The CA-p24 level in the culture supernatant was determined after 48 hours. Average values obtained in three transfections are shown, with the error bars indicating the standard deviation. (B) HeLa X1/6 cells were transfected with the HIV-rtTA variants and the intracellular luciferase level, which reflects rtTA production, was measured after culturing with dox for 48 h. Average values (with standard deviations) are shown for four experiments. (C) Northern blot analysis of the RNA isolated from transfected C33A cells. The position of the 18S and 28S rRNA bands, and the unspliced (9 kb), single spliced (4 kb) and double spliced (2 kb) viral transcript classes are indicated. RNAs with an unexpected size are indicated with a grey triangle. The transcripts that are exclusively observed for the A and B mutants are indicated with an open triangle.

We previously demonstrated that the TAR-destabilizing A and B mutations induce an alternative folding at the 5' end of the HIV-rtTA transcripts. Since TAR is part of the R region that is present at both ends of the viral RNA, the TAR deletions may also affect 3' RNA functions such as polyadenylation. We therefore analyzed the effect of the TAR deletions on viral gene expression, RNA production and processing. C33A cervix carcinoma cells were transfected with the HIV-rtTA molecular clones that contain either the original TAR^m ^hairpin or modified TAR sequences at both the 5' and 3' LTR. After culturing the cells with dox for two days, we quantified virus production by measuring the CA-p24 level in the culture medium (Fig. 2A). CA-p24 production was reduced for the A and B mutants, and it was restored for the AB variant. In addition, we transfected the HIV-rtTA variants into HeLa X1/6 cells, which contain an integrated rtTA/dox-responsive luciferase reporter construct. The luciferase level measured after two days of culturing with dox reflects the production of the virus-encoded rtTA protein. This analysis revealed that rtTA production was also reduced for the A and B mutants and restored to the wild-type level for the AB mutant (Fig. 2B).

Northern blot analysis of RNA isolated from transfected C33A cells revealed that the reduced viral protein production of the A and B mutants correlated with a reduced level of unspliced (9 kb), single spliced (4 kb) and double spliced (2 kb) HIV-rtTA transcripts, whereas normal amounts were observed for the AB mutant (Fig. 2C). Several novel viral transcripts were detected for the A and B mutants, which were not observed with the TAR^m ^and AB viruses (open triangles in Fig. 2C). In addition, RNA molecules with an unexpected size were detected for all virus constructs (grey triangles). Because the viral transcripts were produced from transfected circular HIV-rtTA plasmids, these artificial RNAs may be the product of improper initiation of transcription at the 3' LTR or incomplete 3' LTR polyadenylation of correctly initiated transcripts. Both events will result in the production of odd-size transcripts that comprise vector sequences, which complicates the analysis.

### Destabilization of the 3' TAR element hinders polyadenylation of viral transcripts

**Figure 3:**
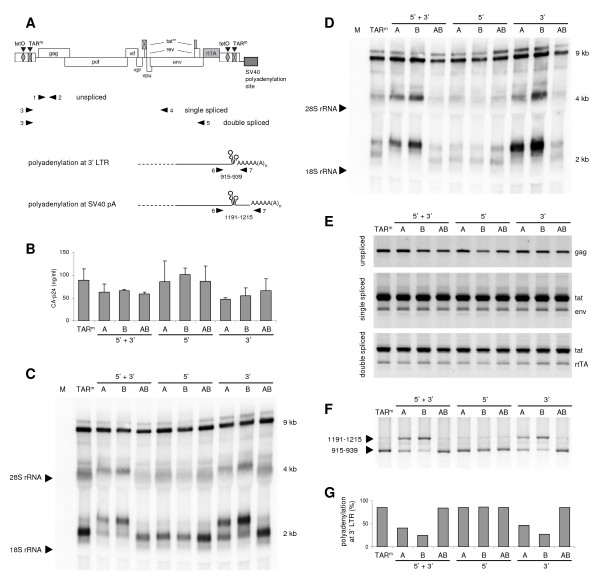
**Destabilization of the 3' TAR hairpin affects polyadenylation**. (A) In the HIV-rtTA-SV40 constructs the SV40 polyadenylation site was placed downstream of the viral genome. The position of the oligonucleotides that were used as primer in the RNA analyses (panels E and F) are indicated. (B) C33A cells were transfected with 5', 3' and 5'+3' mutated constructs and the CA-p24 level in the culture medium was measured after culturing with dox for 48 h. Average values obtained in three transfections are shown, with the error bars indicating the standard deviation. (C) Intracellular RNA was isolated and analyzed by Northern blotting with a probe against the U3/R region of HIV-rtTA. The position of the 18S and 28S rRNA bands, and the unspliced (9 kb), single spliced (4 kb) and double spliced (2 kb) viral transcripts are indicated. (D) The Northern blot was stripped and rehybridized with a probe against the downstream SV40 sequences. Only the extended RNA transcripts observed for the variants with a 3' A or 3' B mutation hybridized with this probe. The residual staining of the normally sized transcripts is due to incomplete stripping of the blot. (E) The isolated RNA was used as template for the production of viral cDNA. The cDNA products were amplified with indicated primers for the unspliced (1+2), single-spliced (3+4) and double-spliced transcripts (3+5). (F) Polyadenylation site usage was analyzed by PCR amplification of the cDNA with primers 6 and 7. Polyadenylation at the 3' LTR results in a 939-bp product, whereas polyadenylation at the SV40 sequence results in a 1215-bp product. For constructs with the A, B and AB deletion in the 3' TAR hairpin, these fragments will be 14, 10 and 24 bp shorter, respectively. The identity of these PCR products was confirmed by sequence analysis. (G) The polyadenylation efficiency at the 3' LTR was calculated by quantification of the 2 kb RNA bands in Fig. 3C.

To avoid inclusion of vector sequences in the viral RNAs, we made a novel set of HIV-rtTA constructs in which the SV40-derived polyadenylation signal is positioned downstream of the 3' LTR (Fig. 3A). Transcripts starting at the 3' LTR of these constructs will be polyadenylated at the SV40 polyadenylation site and such short RNAs will not be detected on the Northern blot. Transcripts starting at the natural 5' LTR promoter that are not polyadenylated at the 3' LTR will be polyadenylated at the SV40 site, which will result in a discrete 276-nt extension. To distinguish 5' LTR from 3' LTR effects, we made a complete set of 5', 3' and 5'+3' TAR mutants.

C33A cells were transfected with the new HIV-rtTA-SV40 constructs. After two days of culturing with dox, the original and all TAR-mutated HIV-rtTA-SV40 constructs showed no significant variation in the production of CA-p24 (Fig. 3B), which contrasts with the reduced protein production of the A and B variants that lacked the SV40 element (Fig. 2A). Analysis of the intracellular RNA by Northern blotting did indeed produce a more standard RNA pattern with only the three major RNA classes (9, 4 and 2 kb) (Fig. 3C). Within the set of 5'+3' TAR mutants an increase in the size of the A and B transcripts was apparent, whereas the size of the AB transcript was similar to that of the original (TAR^m^) virus. This size increase corresponds with what one would expect for read-through transcription to the SV40 polyadenylation site, and was most prominent for the shorter multi-spliced transcripts. The same RNA shift was observed for 3' mutants A and B, but again not for the AB double mutant. To confirm that the longer transcripts are the result of polyadenylation at the SV40 polyadenylation site, the Northern blot membrane was stripped and hybridized with a probe that specifically detects the SV40 sequences present downstream of the 3' LTR. This analysis revealed that the extended transcripts do indeed contain this sequence (Fig. 3D).

To rule out aberrant splicing of the viral transcripts, we analyzed the splice pattern of the TAR-deleted HIV-rtTA-SV40 variants in more detail. The isolated cellular RNA was used for the synthesis of cDNA, which was PCR amplified with primer combinations that detect unspliced or spliced viral RNAs (Fig. 3E). This analysis did not reveal any difference between the original virus and modified variants indicating that the 5' and 3' mutations do not affect splicing. To confirm that the single-side TAR deletions do affect polyadenylation, the 3' end of the viral RNA was further analyzed by 3' RACE (rapid amplification of cDNA ends). The RNA was reverse transcribed using an oligo-dT primer that anneals to the polyA tail and the cDNA product was PCR amplified. For constructs with the original TAR^m ^sequence at the 3' LTR, polyadenylation will result in a PCR product of 939 bp, whereas polyadenylation at the SV40 site will result in a product of 1215 bp (Fig. 3A). For constructs with the A, B and AB deletion in the 3' TAR hairpin, these fragments will be 14, 10 and 24 bp shorter, respectively. A product corresponding to polyadenylation at the 3' LTR was observed for all viruses with a TAR^m ^or AB sequence at the 3' LTR (Fig. 3F, 939-bp product for TAR^m ^and the 5' A, B and AB mutants; 915-bp product for the 3' and 5'+3' AB variants). This demonstrates that these viruses do efficiently polyadenylate at the 3' LTR. In contrast, viruses with a single-side deletion in the 3' TAR element polyadenylated predominantly at the SV40 site, which resulted in the longer PCR product (Fig. 3F, 1201-bp product for 3' and 5'+3' A mutants; 1205-bp product for 3' and 5'+3' B variants). These results demonstrate that the single-side deletions in the 3' TAR element reduce the usage of the adjacent 3' LTR polyadenylation site. Consequently, the downstream SV40 polyadenylation site is used more frequently, resulting in transcripts that are extended by 276 nt. Quantification of the "short" and "long" forms of the double-spliced transcripts on the Northern blot (Fig. 3C) revealed that 80–90% of the viral transcripts with a complete (TAR^m^) or truncated (AB) 3' TAR structure are processed at the natural 3' polyadenylation site, whereas only 30–40% of the transcripts with a single-side deletion in 3' TAR (A, B) are polyadenylated at this position (Fig. 3G).

## Discussion

We demonstrate that destabilization of the TAR hairpin in HIV-1 RNA results in reduced polyadenylation at the 3' end of the viral transcripts. Incomplete polyadenylation of the transcripts will result in read-through transcripts that contain vector or cellular genome sequences when proviral plasmids or integrated proviruses are transcribed, respectively. These improperly polyadenylated transcripts may be less stable and we did indeed observe reduced levels of viral RNA and protein (Fig. 2). The extended viral transcripts may face additional problems in the viral life cycle. For example, they may not be efficiently packaged into virions due to size restriction. Incomplete polyadenylation can thus, at least partially, explain the replication defect of the TAR-destabilized HIV-rtTA variants with a single-side deletion in the TAR stem (A and B mutants). In contrast, the double mutant with a truncated TAR hairpin (AB mutant) demonstrated efficient polyadenylation at the 3' end and viruses with this mutation replicated efficiently [34]. Thus, the TAR hairpin itself is not needed for proper 3' LTR polyadenylation, but TAR destabilization does negatively influence this process.

We recently showed that destabilization of the 5' TAR element does affect the LDI-BMH equilibrium of the leader RNA [35]. Whereas *in vitro *produced wild-type and AB-mutated leader transcripts adopted predominantly the LDI conformation, the A and B mutants demonstrated a shift toward the alternative BMH conformation. Probing of the RNA structure showed that TAR destabilization liberates TAR-nucleotides that can pair with unpaired nucleotides downstream of the polyA hairpin to extend this structure [35]. This extension increases the thermodynamic stability (ΔG) of the polyA hairpin from -17.5 kcal/mole to -24.7 kcal/mole, as predicted by the Mfold program [39]. Stabilization of the 5' polyA hairpin, which is part of the BMH structure, did indirectly affect the LDI/BMH equilibrium and leader-mediated RNA dimerization. Stabilization of the 3' polyA hairpin explains the reduced polyadenylation of the 3'-TAR destabilized transcripts (A and B mutants), as we previously demonstrated that such an increase in the polyA hairpin stability hinders the binding of polyadenylation factors to the AAUAAA polyadenylation signal [40] and decreases the efficiency of polyadenylation [29-31].

It has previously been suggested that folding of the TAR hairpin is important to appropriately space the upstream sequence element (USE) and 3' polyadenylation site in the RNA transcript [20]. This would resemble the situation in the human T-cell leukemia virus type-I (HTLV-1), where folding of a 276-nt spacer functionally juxtaposes the AAUAAA sequence and the polyadenylation cleavage site [41]. The AB-mutation will result in a truncated but stable stem-loop structure that will not affect the spacing between the USE and polyadenylation site. RNA structure probing studies [35] indicated that the A and B mutants fold an alternative, significantly less stable stem-loop structure that may effectively increase the spacer and thus contribute to the reduced polyadenylation efficiency.

Several studies suggested that the TAR hairpin has other functions in HIV-1 replication in addition to its role in transcription, such as in translation, dimerization, packaging and reverse transcription of the viral RNA [9-15,42-44] (and references therein). Most of these studies were complicated by the fact that mutations in TAR have a dominant negative effect on viral transcription, which obscures other effects in the viral life cycle. Using the HIV-rtTA variant that does not need TAR for the activation of transcription, we recently demonstrated that complete deletion of TAR does not abolish *in vitro *replication, which indicates that TAR has no other essential function in HIV-1 replication [34]. Moreover, our TAR deletion studies demonstrate that TAR destabilization is risky because it induces unwanted side effects. TAR opening triggers the formation of an extended and more stable polyA hairpin, which affects the structure and function of both the 5' leader and the 3' end of the viral RNA. These TAR mutations indirectly affect dimerization, packaging and polyadenylation of the viral transcripts, but TAR is not directly involved in these processes. Apparently, the wild-type TAR element is sufficiently stable to prevent the TAR-nucleotides from interacting with other RNA domains.

## Conclusion

Although the TAR hairpin is not directly involved in polyadenylation of the HIV-1 RNA transcripts, destabilization of TAR does affect this process. This study demonstrates that the stability of TAR structure is important for proper folding of the adjacent polyA hairpin.

## Methods

### Construction of HIV-rtTA variants

Construction of the infectious HIV-rtTA molecular clone and variants with a deletion in the 5' TAR or both the 5' and 3' TAR elements were described previously [32,34]. For the construction of the 3' TAR-mutated variants, the BamHI-BglI fragment of the constructs with both 5' and 3' deletions, which encodes the 3' half of the viral genome, was used to replace the corresponding fragment in HIV-rtTA. The SV40 polyadenylation signal was inserted downstream of the 3' LTR in these constructs as follows. We first made a pBlue-NANB cloning vector with NotI, AatII, NcoI and BamHI sites in the multiple cloning site. For this, the primers MCS-NANB-Fw (GGC CGC GAC GTC CAT GGT CTA GAT CTG GAT CCA CGT) and MCS-NANB-Rev (GGA TCC AGA TCT AGA CCA TGG ACG TCG C) were annealed and ligated into the NotI-AatII digested pBluescript fragment of pBue3'LTRext-ΔU3-rtTA_F86Y A209T_-2ΔtetO [32]. The NcoI-BamHI fragment of pGL3-basic (Promega) that encodes the firefly luciferase gene and SV40 polyadenylation site was ligated into the NcoI and BamHI sites of pBlue-NANB, resulting in the pBlue-MCS-luc-SV40pA plasmid. The luciferase gene was removed by digestion with NcoI and XbaI, blunting of the sticky ends with Klenow polymerase and self-ligation of the vector to produce pBlue-MCS-SV40pA. The AatII-BglI fragment of this plasmid, which contains the SV40 polyadenylation signal, was inserted into the AatII and BglI sites downstream of the 3' LTR in the different HIV-rtTA clones.

### Cell culturing

HeLa X1/6 is a HeLa-derived cervix carcinoma cell line containing chromosomally integrated copies of the CMV-7tetO luciferase reporter construct pUHC13-3 [45]. HeLa X1/6 and C33A (ATCC HTB31) [46] were grown as a monolayer in Dulbecco's modified Eagle's medium (DMEM) supplemented with 10% FCS and minimal essential medium nonessential amino acids, penicillin (100 U/ml) and streptomycin (100 μg/ml). All cell cultures were kept at 37°C and 5% CO_2_.

### Transient transfection and RNA isolation

C33A cells were cultured in 10-cm^2 ^wells, grown to 60% confluency and transfected with 5 μg HIV-rtTA construct by calcium phosphate precipitation as previously described [32]. Cells were cultured in the presence of 1 μg/ml doxycycline (dox) (Sigma D-9891) and the culture medium was changed after 16 h. The virus level in the culture medium was quantitated by CA-p24 enzyme-linked immunosorbent assay (ELISA) after two days [47]. The cells were subsequently washed with phosphate buffered saline (PBS), briefly incubated with 0.5 ml 0.05% trypsin-EDTA (Invitrogen) till cells detached from the plate and resuspended in 1 ml 10% fetal bovine serum-containing medium to inactivate trypsin. Cells were pelleted at 2,750 × *g *for 5 min, washed in 1 ml PBS, centrifuged at 2,750 × *g *for 5 min, resuspended in 0.6 ml RLT buffer (QIAGEN) and homogenized with a QIAshredder column (QIAGEN). Total RNA was isolated with the RNeasy kit (QIAGEN) procedure, and contaminating DNA was removed with RNase-free DNase (QIAGEN) that was added during the isolation procedure as described in the RNeasy protocol.

### Northern blot analysis

Gel electrophoresis of RNA was performed on a 1% agarose gel in MOPS buffer (40 mM MOPS, 10 mM sodium acetate, pH 7.0) with 7% formaldehyde at 100 Volt. The RNA was transferred overnight onto a positively charged nylon membrane (Boehringer Mannheim) by means of capillary force. RNA was attached to the membrane with a UV crosslinker (Stratagene). The 373-bp EcoRV-HinDIII fragment of HIV-rtTA encoding the U3/R region was ^32^P-labeled with the High Prime DNA Labeling kit (Roche Diagnostics) and used as HIV-rtTA probe. To generate the SV40 probe, the HIV-rtTA-SV40 TAR^m ^molecular clone was digested with AatII and BamHI and the 276-bp fragment was isolated and labeled as described above. Prehybridization and hybridization was done in ULTRAhyb buffer (Ambion) at 55°C for 1 and 16 h, respectively. The membrane was then washed two times at room temperature for 5 min in low-stringency buffer (2 × SSC, 0.2% SDS) and two times for 10 min at 50°C in high stringency buffer (0.1 × SSC, 0.2% SDS). Images were obtained using the PhosphorImager (Amersham Biosciences) and data analysis was performed with the ImageQuant software package. The Northern blot was stripped by boiling the membrane at 70°C in 0.1% SDS for 3 times 1 h. The stripping efficiency was controlled by scintillation counting and the blot was hybridized with the SV40 probe after prehybridization.

### Splicing and polyadenylation assay

To analyze splicing and polyadenylation of the viral transcripts, the isolated RNA was reverse transcribed with ThermoScript reverse transcriptase at 50°C (Invitrogen), using the oligo(dT)_25 _and random hexamers primers. The cDNA product was used as template in a polymerase chain reaction (PCR) with primers 1 (GAG ACC ATC AAT GAG GAA GCT GCA GAA TGG GA) and 2 (GGC CGG CCC TTG TAG GCC GGC CAG ATC TTC CC) to detect the unspliced RNAs, with primers 3 (TCA ATA AAG CTT GCC TTG AGT GC) and 4 (CTC CGC AGA TCG TCC CAG AT) to detect the single spliced RNAs, and with primers 3 and 5 (CTA TGA TTA CTA TGG ACC ACA CA) to detect the double spliced RNAs. The polyadenylated RNAs were detected with primer 6 (CTG TGT CAG CAA GGC TTC TC) and the 3'RACE adapter primer 7 (GGC CAC GCG TCG ACT AGT ACT TTT TTT TTT TTT TTT T) that anneals to the polyA tail. The cDNA was denatured at 94°C for 5 min and amplified in 35 cycles of 1 min 95°C, 1 min 55°C, 2 min 72°C and a final extension time of 7 min at 72°C. The PCR products were visualized on a 1% agarose gel stained with ethidium bromide.

### rtTA assay

HeLa X1/6 cells were cultured in 2-cm^2 ^wells to 60% confluency and transfected with 1 μg of the HIV-rtTA constructs and 0.5 ng pRL-CMV (Promega), in which the expression of *Renilla *luciferase is controlled by the CMV immediate-early enhancer promoter, to allow correction for differences in transfection efficiency. The cells were cultured with dox (1 μg/ml) and the medium was refreshed after 16 h. The culture medium was collected after 48 hours for CA-p24 measurement. The cells were washed with 1 ml PBS and subsequently lysed with passive lysis buffer (Promega). Firefly and *Renilla *luciferase activities were determined with the dual-luciferase assay (Promega). The rtTA level was calculated as the ratio between the firefly and *Renilla *luciferase activities and corrected for between session variation [48].

## Competing interests

The authors declare that they have no competing interests.

## Authors' contributions

MMV and AH performed the experiments. MMV drafted the manuscript. ATD and BB designed the experiments and revised the manuscript.
